# Combined Effects of Ethnicity and Education on Burden of Depressive Symptoms over 24 Years in Middle-Aged and Older Adults in the United States

**DOI:** 10.3390/brainsci10040209

**Published:** 2020-04-02

**Authors:** Shervin Assari

**Affiliations:** Department of Family Medicine, Charles R. Drew University of Medicine and Science, Los Angeles, CA 90095, USA; assari@umich.edu; Tel.: +1-734-232-0445; Fax: +1-734-615-8739

**Keywords:** socioeconomic position, socioeconomic status, educational attainment, race, ethnic groups, Latino, Hispanic

## Abstract

Ethnicity and educational attainment are among the major social determinants of depression in the general population. While high education credentials protect individuals against depressive symptoms, this protection may be weaker for ethnic minority groups such as Hispanic Whites compared to the majority group (non-Hispanic Whites). Built on marginalization-related diminished returns (MDRs), the current study used 24-year follow-up data from a nationally representative sample of middle-aged and older adults to explore ethnic variation in the protective effect of education levels against the burden of depressive symptoms over time. Data for this analysis were borrowed from the Health and Retirement Study (HRS 1992–ongoing), a nationally representative longitudinal study. HRS followed 8314 middle-aged and older adults (50+ years old) for up to 24 years. From this number, 763 (9.2%) were Hispanic White, and 7551 (90.8%) were non-Hispanic White Americans. Education level was the independent variable. We had two outcomes. Firstly, using cluster analysis, individuals were categorized to low- and high-risk groups (regarding the burden of depressive symptoms over 24 years); secondly, average depressive symptoms were observed over the 24 years of follow up. Age and gender were the covariates. Ethnicity was the moderator. Linear and logistic regression were used for analysis. Logistic regression showed that, overall, high educational credentials reduced the odds of chronic depressive symptoms over the 24 years of follow-up. Linear regression also showed that higher years of education were associated with lower average depressive symptoms over time. Both models showed statistically significant interactions between ethnicity and graduation, indicating a smaller protective effect of high education against depressive symptoms over the 24 years of follow-up time among Hispanic with respect to non-Hispanic White people. In line with the MDRs, highly educated Hispanic White Americans remain at high risk for depressive symptoms, a risk that is unexpected given their education. The burden of depressive symptoms, however, is lowest for highly educated non-Hispanic White Americans. Policies that exclusively focus on equalizing educational gaps across ethnic groups may fail to eliminate the ethnic gap in the burden of chronic depressive symptoms, given the diminished marginal health return of education for ethnic minorities. Public policies must equalize not only education but also educational quality across ethnic groups. This aim would require addressing structural and environmental barriers that are disproportionately more common in the lives of ethnic minorities across education levels. Future research should test how contextual factors, residential segregation, school segregation, labor market practices, childhood poverty, and education quality in urban schools reduce the health return of educational attainment for highly educated ethnic minorities such as Hispanics.

## 1. Introduction

Socioeconomic status is among the significant determinants of mental health outcomes [1,2,3,4,5], including but not limited to clinical depression [6,7,8,9,10,11] and depressive symptoms [6,12,13,14]. Individuals with higher educational attainment are protected against the risk of mental health problems [1,2] such as depression [14,15,16]. These studies are, however, predominantly cross-sectional or, if longitudinal, they have short follow-up periods. In addition, very little is known about the relevance of socioeconomic status (SES), particularly education, to the mental health of middle-aged and older adults.

High SES, however, differently impacts the mental and physical health of sub-populations [17,18]. For example, education [19], employment [20,21], marital status [22], and income [23] show smaller health effects for ethnic minorities than non-Hispanic White individuals. As a result, middle-class ethnic minorities [23] may remain at high risk of health risk behaviors [24] and depression [25,26,27,28,29,30]. Whether these diminished returns of education on various health outcomes remain in middle-aged and older adults and whether these patterns remain the same over a long period are still unclear [31]. Most of this literature is on the comparison of non-Hispanic Whites and non-Hispanic Blacks, and less is known about comparisons between Hispanics and non-Hispanics [32,33,34]. Moreover, almost nothing is known on how Hispanic and non-Hispanic older adults vary in the long-term effects of education on the burden of depressive symptoms.

### 1.1. Marginalization-Related Diminished Returns (MDRs)

According to the MDRs [17,18], health effects of SES indicators, particularly educational attainment, are systematically weaker on the members of socially marginalized groups in comparison to the members of socially privileged groups. This is mainly established for the comparison of non-Hispanic Blacks relative to Whites (the majority group). Research shows that educational attainment generates worse employment conditions and lower income [1,2,35] [21,36] for the members of ethnic minority groups compared to non-Hispanic Whites. Education attainment also better reduces environmental exposures for non-Hispanic Whites than ethnic minorities [21,37]. As a result, middle-class ethnic minority individuals who have access to high education [38], employment [39], marital status [40], and income [23] remain at risk of mental health problems. 

As a result of these MDRs, highly educated ethnic minorities remain at risk of low exercise [41], unhealthy diet [42], alcohol use, poor school performance [24], aggression [24], tobacco use [24], health service use [43], stress [20,21,30], anxiety [22], depression [26,44], and suicide [45]. As a result, the health of middle-class ethnic minority groups still suffers despite having access to SES resources. It was shown that the effects of various SES indicators such as education [26,46], marital status [40], and employment [47] on various health outcomes are all weaker for ethnic minorities than non-Hispanic Whites. Similarly, education better reduces the risk of hospitalization [33] and disability [48] for non-Hispanic Whites than ethnic minorities. Similarly, education and income better reduce the risk of depression [26] for non-Hispanic Whites than ethnic minorities. It is, however, unknown if these effects remain stable over long periods. Furthermore, while most research is done on adolescents and younger adults, there is a need to test whether such patterns hold up among older adults.

### 1.2. Mechanisms of MDRs

Many potential explanations exist for why education shows smaller health effects for ethnic minorities than non-Hispanic Whites. Poor quality of education in predominantly minority communities is one explanation for the MDRs of education for these populations [49,50,51,52]. Manly and colleagues suggested that poor quality of schooling and education may partially explain some of the ethnic differences in the protective effects of education on conditions such as Alzheimer’s disease [51,53,54,55,56]. We also found that education and education quality, as well as school bonding [57] and educational mobility [19,58,59], may all have some role in this phenomenon.

Another explanation of the MDRs phenomenon is the labor market discrimination of ethnic minorities [36,60]. MDRs of education on health may be because of the MDRs of education in the labor market in generating income and wealth [35,61,62]. We know that high education level has a smaller effect on reducing the risk of poverty among ethnic minorities, compared to non-Hispanic White Americans [35,61]. As a result of labor market discrimination, highly educated ethnic minorities work in low-quality and stressful jobs [21,62]. Such conditions increase the environmental exposure of highly educated ethnic minorities with respect to non-Hispanic White people to environmental exposures with serious health hazards [21,37,60,63]. 

Another mechanism is interpersonal discrimination. High-SES ethnic minorities face and report more, not less discrimination [9,13,14,64]. High SES also increases vulnerability to discrimination [65]. In a study, high-SES ethnic minorities were more likely to develop depression as a result of discrimination [65]. This might be, in part, because highly educated people are more likely to be surrounded by White people, across all ethnic groups. This increased exposure to White people increases their exposure to perceived discrimination and prejudice [28,29,66,67]. 

### 1.3. Aims

To better understand whether the MDRs phenomenon also applies to ethnic disparities in the burden of depressive symptoms over a long period, we compared non-Hispanic and Hispanic White middle-aged and older Americans for the protective effect of education level on chronic depressive symptoms over 24 years of follow-up. Although research thoroughly documented the additive effects of ethnicity and SES (education level) on long-term health outcomes [68,69], very few studies ever tested MDRs of educational attainment on morbidity over a long period of time [70]. Such a study would require studying the non-additive and multiplicative effects of ethnicity and educational attainment on long-term data on various aspects of health. Such an approach would require long-term data with follow-up of health over a long period. We used data from the Health and Retirement Study (HRS), an ongoing study with a national and representative sample of middle-aged and older adults (50–59 years old at baseline). In line with the MDRs [17,18], we expected smaller protective effects of education level on the burden of depressive symptoms over time for Hispanic compared to non-Hispanic White Americans. 

Built on the Health and Retirement Study (HRS) data, this study explored ethnic variation in the effects of educational attainment and income on a 24-year life expectancy of middle-aged and older American adults. In concordance with the MDRs framework [17,71], we expected weaker effects of baseline educational credentials on the long-term burden of depressive symptoms of Hispanic White middle-aged and older adults in comparison to their non-Hispanic White counterparts. In other terms, we expected a disproportionately high level of chronic depressive symptoms in highly educated Hispanic White middle-aged and older adults. However, we expected the lowest level of burden of depressive symptoms over time for highly educated non-Hispanic White middle-aged and older adults.

## 2. Methods

### 2.1. Design and Setting

Data came from the first 10 waves of the Health and Retirement Study (HRS, 1992–ongoing). The HRS is a long-term longitudinal study, with biannual repeated measurements. The study recruited and followed a nationally representative sample of American middle-aged and older adults (age 50–59 years at baseline). Although more detailed methodological information exists on study design, measures, sample, and sampling of the HRS [72], we provide a summary of the critical aspects of the HRS.

### 2.2. Sample and Sampling

The HRS used a national area probability sampling to recruit participants. The current analysis only included the core (primary) sample that was recruited in 1992. All participants were 50–59 years old at wave one. This means that all our participants were born between 1931 and 1941. The HRS sample is representative of all 50–59-year-old middle-aged and older adults who resided in United States (US) households in 1992 (Wave 1). 

### 2.3. Analytical Sample

The analytical sample of the current study involved 50+ years old Americans who were non-Hispanic or Hispanic White. Only White people were enrolled in our analysis; thus, all Black people were excluded. All participants in the HRS core sample could enter our analysis regardless of the duration of follow-up because our outcome was time from follow-up to mortality. Thus, participants were analyzed only if they were non-Hispanic or Hispanic White (*n* = 8314).

### 2.4. Data Collection

The HRS study collected data that extended to various aspects of the participants. This data collection included demographic information, SES indicators, social factors, psychological assets and traits, health behaviors, health service utilization, and objective and subjective measures of health. While HRS collected data from both participants and their spouses, we only used the participants’ data. Data were collected biannually. The HRS data were collected via either telephone or a face-to-face interview. For individuals who were not available themselves, a proxy interview was used. 

### 2.5. Measures

*Demographic Factors.* Age (years) and gender (0/1) were demographic factors. Age was a continuous measure (recorded as the number of years passed since birth). Gender was a dichotomous variable, coded as male = 1, female = 0. Age and gender were both measured in 1992. 

*Socioeconomic Status (SES) Indicator.* In this study, educational attainment was treated in two ways: firstly, as a categorical variable: (1) less than high-school diploma (reference group), (2) high-school diploma, (3) some college, and (4) college graduate; secondly, as a continuous measure. For education as a continuous measure, we used years of schooling, which was measured at baseline (1992). The categorical variable was entered into our logistic regressions, and the continuous variable was entered into our linear regressions.

*Depressive Symptoms.* HRS used the eight-item Center for Epidemiological Studies Depression (CES-D) measure to assess depressive symptoms. Items were on a 0/1 response scale. Depressive symptoms were measured every two years, from 1994 to 2016. For this variable as a continuous measure, we calculated the mean score over time. For this measure as a categorical/binary variable, we used k-mean cluster analysis to define risk categories over the 24 years of follow-up (the first wave did not have depressive symptoms). The categorical variable was entered into our logistic regressions, and the continuous variable was entered into our linear regressions.

### 2.6. Data Analysis 

We analyzed the HRS data using SPSS 23.0 (IBM Corporation, Armonk, NY, US). For our univariate analyses, means (standard deviation: SD) and absolute/relative frequencies (*n* and %) were reported. We calculated two outcomes: firstly, a binary (categorical) variable, and secondly, a continuous measure. To define our binary (categorical) outcome, we used k-mean cluster analysis with all observations of depressive symptoms (*n* = 12) as input variables. Using this approach, participants were categorized as low-risk (coded as 0) or high-risk (coded as 1) groups. Our continuous outcome was mean depressive symptoms over the follow-up period. For multivariable analysis of our binary (categorical) outcome, we applied logistic regressions. For our continuous measure, we ran linear regression models. Our modeling strategies remained similar; however, years of education and educational credentials were the outcomes for linear regression and logistic regression, respectively. In these models, membership of the cluster of chronic depressive symptoms was considered as binary outcomes. Firstly, we ruled out multi-collinearity between ethnicity and educational attainment. We did not have any missing data for our outcomes because individuals could become a member of a cluster group regardless of the number of observations and missing data. We ran 12 logistic regression models, three for each outcome: *Model 1* was without interaction in the pooled sample, *Model 2* was with the statistical interaction terms between ethnicity and educational attainment in the pooled sample, *Model 3* was in non-Hispanic White people, and *Model 4* was in Hispanic White people.

### 2.7. Ethics Statement

The HRS study protocol was approved by the University of Michigan (UM) Institutional Review Board (IRB). All HRS participants signed written consent. The data were collected, stored, managed, and analyzed in a fully anonymous fashion. As we used fully de-identified publicly available data, this study was non-human subject research, according to the National Institutes of Health (NIH) definition.

## 3. Results

### 3.1. Descriptives

This analysis included a total of 8314 middle-aged and older adults (50+ years old) who were followed for 24 years. From this number, 763 (9.2%) were Hispanic White, and 7551 (90.8%) were non-Hispanic White Americans. 

Table 1 presents a summary of the descriptive characteristics of the overall sample and by ethnicity. As this table shows, Hispanics had lower education but were more likely to be in the chronic depressed group. They also had higher average depressive symptoms over the 24 years of follow-up.

### 3.2. Logistic Regressions

Table 2 summarizes the results of two logistic regressions with chronic depressive symptoms over 24 years as the outcome. *Model 1-a* was without interactions (main effects model), while *Model 2-a* (main effects + interaction model) had statistical interactions between levels of educational attainment and ethnicity. *Model 1-a* showed that, overall, higher education credentials are associated with lower odds of chronic depressive symptoms over the 24 years of follow-up, while all confounders were controlled. *Model 2-a* showed a statistically significant interaction between the effects of ethnicity and college graduation on the outcome, suggesting that the protective effect of college graduation against the risk of chronic depressive symptoms was smaller for Hispanic White than non-Hispanic White Americans.

Table 3 also summarizes the results of two identical ethnic-specific logistic regression models. *Model 3-a* was performed in non-Hispanic White Americans, and *Model 4-a* was performed in Hispanic White Americans. *Model 3-a* and *Model 4-a* showed that college graduation was associated with lower odds of chronic depressive symptoms over 24 years for non-Hispanic White but not Hispanic White Americans (Table 3).

### 3.3. Sensitivity Analysis

To run some sensitivity analyses, we also ran linear regression models with mean depressive symptoms over the 24 years of follow-up as the outcome. Our modeling strategy stayed the same as our main logistic regression models. Firstly, we ran two linear regressions with mean depressive symptoms over 24 years as the outcome. *Model 1-b* was without interactions (main effects model), while *Model 2* (main effects + interaction model) had a statistical interaction between years of schooling (educational attainment) and ethnicity. *Model 1* showed that, overall, a higher number of years of schooling (educational attainment) was associated with lower mean depressive symptoms over the 24 years of follow-up, while all confounders were controlled. *Model 2-b* showed a statistically significant interaction between the effects of ethnicity and years of schooling on the outcome, suggesting that the protective effect of higher number of years of schooling against mean depressive symptoms over time was smaller for Hispanic White than non-Hispanic White Americans. We also ran two identical ethnic-specific linear regression models. *Model 3-b* was performed in non-Hispanic White Americans, and *Model 4-b* was performed in Hispanic White Americans. *Model 3-b* and *Model 4-b* showed that a higher number of years of schooling was associated with lower mean depressive symptoms over 24 years for non-Hispanic White and Hispanic White Americans; however, the magnitude of this protection was larger for non-Hispanic White than Hispanic White Americans (Table 4 and Table 5).

## 4. Discussion

This study documented two main findings. Firstly, high education level at baseline was associated with better health status over 24 years of follow-up in the overall sample. Secondly, compared to non-Hispanic White Americans, Hispanic White Americans remained at a relative disadvantage regarding the protective effects of education level on various aspects of health. That is because the inverse association between education level and various aspects of morbidity is weaker for Hispanic than non-Hispanic White middle-aged and older Americans.

The first finding is an extension of previous research. While we know that population health improves as education level and SES improve, most of the literature on social determinants of health and social gradient in health was conducted using a cross-sectional design or, if longitudinal, a short-term follow-up period. The protective health effect of SES, particularly education level, is well established in the literature [73,74,75]. The social gradient of health is well known [76,77]. Each additional year of schooling has a protective effect on health [73,74]. Similarly, educational credentials have some protective effects [73,74]. Overall, morbidity and mortality are more common at the bottom of society, which is closely linked to SES and education [78,79,80,81,82,83].

The second finding is also an extension of what we knew from cross-sectional or short-term follow-up studies. Studies by Navarro [84,85,86] and Farmer and Ferarro [87] mentioned that it is ethnicity and SES, but not ethnicity or SES, that cause disparities. Our previous research also documented the considerably weaker impact of education and other SES indicators on the well-being of ethnic minorities other than non-Hispanic White Americans [17,71]. In various studies, education more strongly improved overall health [32,62,88], impulsivity [89], drinking [46,90], smoking [91,92], sleep [93], exercise [41], suicide [45], and depression [6,28,44] for non-Hispanic White Americans than ethnic minority Americans. These patterns are robust, as shown for Hispanics [94], non-Hispanic Blacks [95,96], Native Americans [36], East Asians [97,98], and members of the LGBT community [99]. This universal pattern suggests that MDRs are not due to the group or individual behaviors but due to structural and societal processes that hinder individuals’ and populations’ ability to leverage their education and other SES indicators [17,71]. Although these patterns are seen for all non-privileged groups, they are more robust for Blacks than Hispanics or other ethnic groups.

The unique contribution of this study is that it extends previous findings. Some unique advantages include a nationally representative sample, a large sample, and a very long follow-up period. We also applied two statistical approaches to increase the robustness of our findings. While considerable research previously showed MDRs of education and income on a wide range of mental health outcomes, this research mainly involved cross-sectional or short-term follow-up studies. This study was unique in reporting 24 years of depressive symptoms as the outcome.

### 4.1. Limitations

Each study has its limitations. The current study is not an exception to that rule. To list the significant methodological limitations of our study, we can refer to the measurement of depressive symptoms. Depressive symptoms were measured using self-reported data. We also did not control for wealth. Thus, our study was at risk of omitted confounders. This study only included area-level SES indicators. Another limitation was missing confounders such as wealth, income, employment, and marital status. Finally, a limitation in this study was how CES-D response items were applied. While CES-D uses four-item responses, the HRS uses 0/1 responses, which reduces the validity and reliability of the measure.

### 4.2. Future Research

Future data may validate self-reported depressive symptoms using triangulation methods such as clinical diagnosis by a psychiatrist, administrative data, chart review, or claim data. Future research may test if these results can be replicated in other datasets and settings. There is also a need to replicate these findings using other SES indicators. There is a need to test MDRs in other ethnic and racial groups, immigrants, and other marginalized people such as members of the LGBT community.

## 5. Conclusions

In the United States, the inverse associations between education level and burden of depressive symptoms over time are weaker for Hispanic White than non-Hispanic White middle-aged and older Americans. Future research may investigate contextual factors that explain why middle-class ethnic minorities remain at risk of depressive symptoms and poor mental health.

## Figures and Tables

**Table 1:** Descriptive statistics (*n* = 8314).

Characteristics	All		Non-Hispanic Whites		Hispanic Whites	
	**Mean**	**SD**	**Mean**	**SD**	**Mean**	**SD**
Age (years)	56.42	4.43	56.43	4.38	56.34	4.83
Average depressive symptoms over time *	1.38	1.52	1.29	1.44	2.24	1.92
						
	***N***	**%**	***N***	**%**	***N***	**%**
**Ethnicity**						
Non-Hispanic	7551	90.8	7551	100.0	-	-
Hispanic	763	9.2	-	-	763	100.0
**Gender**						
Female	4132	49.7	3733	49.4	399	52.3
Male	4182	50.3	3818	50.6	364	47.7
Education *						
Less than high-school diploma	1893	22.8	1422	18.8	471	61.7
High-school diploma	3276	39.4	3118	41.3	158	20.7
Some college	1632	19.6	1536	20.3	96	12.6
College graduate	1513	18.2	1475	19.5	38	5.0
High depressive symptoms *						
No	6609	79.5	6149	81.4	460	60.3
Yes	1705	20.5	1402	18.6	303	39.7

* *p* < 0.05, standard deviation (SD).

**Table 2:** Pooled sample and ethnic-specific logistic regressions of depressive symptoms over 24 years. OR—odds ratio; CI—confidence interval.

	Model 1-*a* (Main Effects)				Model 2-*a* (Main Effects + Interactions)			
	OR	95% CI		*p*	OR	95% CI		*p*
Ethnicity (Hispanic) *	1.85	1.56	2.19	<0.001	0.70	0.62	0.78	<0.001
Gender (male) *	0.70	0.62	0.78	<0.001	1.63	1.31	2.01	<0.001
Age (years)	1.00	0.99	1.01	0.935	1.00	0.99	1.01	0.931
Education				<0.001				<0.001
Less than high-school diploma	1.00				1.00			
High-school diploma *	0.44	0.38	0.50	<0.001	0.42	0.36	0.48	<0.001
Some college *	0.33	0.28	0.39	<0.001	0.32	0.26	0.38	<0.001
College graduate *	0.20	0.16	0.24	<0.001	0.18	0.15	0.23	<0.001
Education × ethnicity								0.062
High-school diploma × ethnicity	-	-	-	-	1.28	0.85	1.92	0.244
Some college × ethnicity	-	-	-	-	1.30	0.77	2.20	0.320
College × ethnicity *	-	-	-	-	2.65	1.24	5.66	0.012
Intercept	0.58			0.149	0.60			0.175
								

OR = Odds Ratio, CI = Confidence Interval, * *p* < 0.05.

**Table 3:** Ethnic-specific logistic regressions for chronic depressive symptoms as the outcome.

	Model 3-a Non-Hispanic				Model 4-a Hispanic			
	OR	95% CI		*p*	OR	95% CI		*p*
Gender (male) *	0.72	0.64	0.82	<0.001	0.55	0.41	0.75	<0.001
Age (years)	1.00	0.99	1.02	0.735	0.99	0.96	1.02	0.638
Education				<0.001				<0.001
Less than high-school diploma	1.00				1.00			
High-school diploma *	0.42	0.36	0.49	<0.001	0.54	0.36	0.79	0.002
Some college *	0.32	0.26	0.38	<0.001	0.41	0.25	0.67	<0.001
College graduate *	0.18	0.15	0.23	<0.001	0.49	0.24	1.02	0.058
Intercept	0.53			0.122	1.74			0.550

OR = Odds Ratio, CI = Confidence Interval, * *p* < 0.05.

**Table 4:** Pooled sample linear regressions for mean depressive symptoms over 24 years as the outcome.

		Mode 1-b				Mode 2-b		
	b	95% CI		*p*	b	95% CI		*p*
Ethnicity (Hispanic) *	0.41	0.29	0.52	0.000	−0.08	−0.34	0.19	0.576
Gender (male) *	−0.32	−0.38	−0.25	0.000	−0.32	−0.38	−0.25	0.000
Age (years)	0.00	0.00	0.01	0.384	0.00	0.00	0.01	0.387
Education *	−0.13	−0.14	−0.12	0.000	−0.14	−0.15	−0.13	0.000
Education × ethnicity *					0.05	0.03	0.08	0.000
Intercept *	2.90	2.46	3.33	0.000	3.04	2.61	3.48	0.000

CI = Confidence Interval, **b** = unstandardized regression coefficient, * *p* < 0.05.

**Table 5:** Ethnic-specific linear regressions for mean depressive symptoms over 24 years as the outcome.

		Model 3-b Non-Hispanic			Model 4-b Hispanic			
	b	95% CI		*p*	b	95% CI		*p*
Gender (male) *	−0.27	−0.34	−0.21	0.000	−0.73	−1.00	−0.46	0.000
Age (years)	0.00	0.00	0.01	0.209	−0.01	−0.04	0.02	0.625
Education *	−0.14	−0.15	−0.13	0.000	−0.09	−0.12	−0.06	0.000
Intercept *	2.94	2.49	3.38	0.000	3.72	2.11	5.33	0.000

CI = Confidence Interval, **b** = unstandardized regression coefficient, * *p* < 0.05.
